# Intelligent Biopolymer-Based Films: Promising New Solutions for Food Packaging Applications

**DOI:** 10.3390/polym16162256

**Published:** 2024-08-08

**Authors:** Diana Ionela Dăescu, Diana Maria Dreavă, Anamaria Todea, Francisc Peter, Iulia Păușescu

**Affiliations:** Faculty of Industrial Chemistry and Environmental Engineering, University Politehnica Timișoara, 300001, Vasile Pârvan 6, 300001 Timișoara, Romania; diana.daescu@student.upt.ro (D.I.D.); diana.dreava@upt.ro (D.M.D.); anamaria.todea@upt.ro (A.T.); francisc.peter@upt.ro (F.P.)

**Keywords:** food packaging, intelligent films, anthocyanidin derivatives, food quality control

## Abstract

The development of biopolymer-based films represents a promising direction in the packaging industry that responds to stringent needs for sustainability, reducing the ecological impact. Traditional fossil-derived polymers present major concerns because of their long decomposition time and their significant contribution to the pollution of the environment. On the contrary, biopolymers such as chitosan, PVA, and PLA offer viable alternatives. This study aimed to obtain an innovative pH indicator for smart packaging using a synthetic non-toxic anthocyanin analogue dye incorporated in bio-based films to indicate meat freshness and quality. The pH-responsive color-changing properties of the dye make it suitable for developing intelligent films to monitor food freshness. The obtained polymeric films were characterized by FT-IR and UV–VIS spectroscopy, and their thermal properties were assessed using thermogravimetric methods. Moisture content, swelling capacity, and water solubility of the polymeric films were also evaluated. The sensitivity of the biopolymer–flavylium composite films to pH variations was studied in the pH range of 2 to 12 and noticeable color variations were observed, allowing the monitoring of the meat’s quality damage through pH changes. The pH-responsive films were applied directly on the surface or in the proximity of pork and chicken meat samples, to evaluate their colorimetric response to fresh and spoilt meat. This study can be the starting point for creating more durable packaging solutions leading to a circular economy.

## 1. Introduction

Petroleum-based plastics such as polyethylene, polypropylene, polyester, and polyamides have good elasticity and heat resistance and are also cheap to use. However, they raised environmental concerns because they produce microplastics that are very difficult to degrade. Due to these concerns along with those related to their health, consumers moved from petroleum-based to biodegradable packages [1,2].

Food waste also represents an issue today because annually approximately 1.3 billion tons of food is lost or wasted throughout the supply chain, from production to consumption [3]. The World Health Organization reported that 420,000 people die annually from diseases caused by food contamination [4]. Oxidation, microbial growth, and decomposition of enzymes are the processes that frequently produce food spoilage, affecting both the economy and the health of consumers [5]. For this reason, packaging optimization strategies have been developed: varying the dimensions of packages to help consumers buy the appropriate quantity of food and proposing packages that preserve the quality of foods and extend their shelf life [6].

The reasons mentioned above led to the conclusion that monitoring the quality of food is necessary for two reasons: to protect consumers from diseases resulting from food spoilage and to increase the productivity of the industry. Intelligent packages are developed to detect storage conditions, quality, and shelf life, to track microbial growth, and to monitor the freshness of the food [7]. Active packages are used to improve the safety, quality, and shelf life of products by absorption or discharge from or in packaged food [8].

Bio-based polymers are mainly used to provide a matrix, where pigments and bacteriostatic agents can be incorporated, and can be classified into four types: polysaccharides, proteins, lipids, and composite polymer carriers [9]. The characteristics that these polymers must achieve in pH-responsive indicator films are low water sensitivity, colorless, good film-forming properties, non-toxic, mechanical, and barrier properties, as well as the ability to catch pigments [5,10].

A smart system that provides visual information on food spoilage, which is frequently linked to a change in pH, mainly uses chromogenic indicators to make the connection with the freshness of the product [11,12]. There was an increased interest in bio-based pH indicator systems such as starch/PVA (polyvinyl alcohol) matrix [1,13], pullulan/chitosan matrix [10], cornstarch/chitosan matrix [14], chitosan/PVA matrix [15], methylcellulose/chitosan nanofiber matrix [16], chitosan [2], starch/gelatin matrix [17], PLA (polylactic acid) [18], PLA/cellulose [19], PLA/PCL [20], PLA/starch [21], PLA/PEG [22]. 

Chitosan is a natural, non-toxic, biodegradable, biocompatible polymer resulting from the partial deacetylation of chitin which possesses antioxidant, antimicrobial, and anticancer properties [23,24,25]. Chitosan is often used in blends with other polymers to combine their benefits and minimize some disadvantages, such as low thermal and mechanical stability and high moisture sensitivity [24]. 

PVA is a biodegradable, nontoxic, biocompatible, and water-soluble synthetic polymer widely used in biomedical and pharmaceutical applications, as well as in food packaging composites [26].

PLA is a natural, biodegradable, and biocompatible polymer obtained by polymerization of lactic acid from natural sources, which is easy to mold and can be decomposed into water and carbon dioxide, being also not harmful to the environment [18,27]. 

Blending chitosan with PVA was found to be a favorable method for enhancing the mechanical and physicochemical properties of chitosan [28]. PVA is able to form clear films and has satisfactory physicochemical properties such as chemical resistance and barrier properties [28,29]. In food packaging, it showed excellent flexibility, good film formation capacity, and high compound cohesion [30].

Anthocyanins represent a group of water-soluble pigments used in nutraceuticals, pharmaceuticals, and food production. Cyanidin, delphinidin, pelargonidin, peonidin, malvidin, and petunidin are among the most common anthocyanins found in fruits and vegetables such as red grape, blackcurrant, elderberry, blackberry, raspberry, black chokeberry, red cabbage, black carrot, purple corn, red radish, and purple-fleshed sweet potato [5,31,32]. Anthocyanins that are simple to integrate into eco-friendly polymers have been widely used for their pH-responsive color-changing properties as freshness indicators in bio-polymer-based films, applied mainly to pork meat, fish, shrimp, and milk products [33,34]. Due to their chemistry and structure, they are also sensitive to other external factors such as light and temperature [35]. These sensitivities, mainly light and temperature, limit their use as indicators of chromogenic freshness [36]. Moreover, the extraction, purification, and separation of anthocyanins involve high costs and arduous work [37]. To reduce these disadvantages, synthetic dyes inspired by natural pigments such as anthocyanins have been studied to improve the necessary properties through low-cost and efficient methods [36,38]. Although the research accomplished in this area is scarce, the importance of the topic definitely justifies the investigation of new bio-inspired flavylium derivatives that could be efficient in replacing natural anthocyanins as possible food quality monitoring compounds in combination with non-toxic and biodegradable eco-friendly polymers. 

The aim of this study was to obtain innovative pH indicators for smart packaging by producing bio-based films with a selected anthocyanin analog dye used for the first time for possible authentication of the food quality. Another important novelty of this research is the evaluation of the efficiency of different biopolymer-flavylium dye film compositions on chicken and pork meat samples. The results demonstrated that they are accurate and reliable in indicating pH changes; thus, they can monitor food freshness. Chitosan, PLA, and polyvinyl alcohol (PVA) were chosen as polymeric matrices because of their desirable properties and wide applications in different sectors, as previously stated.

## 2. Materials and Methods

### 2.1. Materials

The flavylium derivative 6-hydroxy-4′-hydroxy-3′,5′-dimethoxyflavylium hydrogensulfate was synthesized and purified as previously described [38]. Chitosan (low molecular weight, deacetylation degree ≥ 75%), trisodium phosphate (Na_3_PO_4_, 96%), citric acid (≥99.5%), 2,2-diphenyl-1-picrylhydrazyl (DPPH), and chloroform (≥99%) were purchased from Sigma Aldrich (Steinheim am Albuch, Germany). Polylactic acid (PLA, Mw ~60000 Da) was acquired from GoodFellow (Huntingdon, UK). Polyvinyl alcohol (PVA, Mw 89000–98000 Da, >99% hydrolyzed) and acetic acid (CH_3_COOH, 98%) were acquired from Merck KGaA (Darmstadt, Germany), glacial acetic acid (CH_3_COOH, 100%) and ethanol (EtOH, ≥96%) were purchased from CHIMREACTIV SRL (Bucuresti, Romania). Boric acid (H_3_BO_3_, ≥99.5%) was acquired from VWR Life Science (Columbus, OH, USA), and methanol (MeOH, ≥99.98%) from VWR Chemicals BDH (Fontenay-sous-Bois, France). All reagents were used without any purification.

### 2.2. Methods

Preparation of the polymer–dye composite films has been carried out in duplicate, showing excellent reproducibility. All experiments concerning the properties of the polymer–dye composites were performed in triplicate, except the antioxidant activity, which was performed in duplicate. 

#### 2.2.1. Film Preparation

- Chitosan-based films

The films containing chitosan were prepared according to Yoshida et al. [39] with some adjustments by dissolving 1 g chitosan in 50 mL acetic acid 2% and by adding 10 mg of flavylium dye, then stirred magnetically at room temperature for 2 h. Similarly, a film without dye was prepared [39]. Both mixtures were sonicated for CO_2_ removal, then carefully poured into Petri plates with a diameter of 15 cm and dried in an oven for 72 h at 40 °C.

- Chitosan–PVA-based films

Chitosan and PVA stock solutions were prepared by dissolving 0.6 g chitosan in 30 mL acetic acid 2% and 1.4 g PVA in 70 mL water at 70 °C, respectively, using a previously described method [40], with some modifications.

The film was prepared by mixing 15 mL chitosan solution with 35 mL PVA solution and adding 10 mg of flavylium dye. Similarly, a film without adding dye was obtained to be used as a reference, as described [40], with some adjustments. Both solutions were sonicated for 15 min to remove CO_2_, carefully poured into Petri plates of 15 cm diameter, and dried in an oven at 40 °C for 72 h.

- PVA-based films

The third film was obtained by dissolving 1 g PVA and 10 mg of flavylium dye in 50 mL distilled water followed by magnetic stirring at 70 °C until total solubilization. Following the same procedure, a film without dye was obtained as indicated in the literature [40], with some modifications. Both solutions were placed in an ultrasonic bath to remove CO_2_, then carefully poured into Petri plates with a diameter of 15 cm and dried in an oven for 72 h at 40 °C.

- PLA-based films

Using an adjusted method described by Lu R. et al. [41], the fourth film was prepared by dissolving 1 g PLA and 10 mg of flavylium dye in 50 mL chloroform followed by magnetic stirring until total solubilization. Similarly, a film without adding dye was obtained [41]. Both solutions were subjected to ultrasonication for 15 min, then carefully poured into Petri vessels of 15 cm diameter and dried in an oven for 72 h at 40 °C.

#### 2.2.2. Evaluation of Halochromic Properties

Buffer solutions were prepared following a procedure previously described [42], and pH values were measured with a Mettler Toledo Seven Compact S210-K pH meter (Mettler Toledo, Columbus, OH, USA) at 25 °C. For this study, 1 cm × 1 cm polymeric films with dye pieces were immersed in buffer solutions ranging from 2 to 12 (10 mL), and after 2 h they were removed from the solutions, wiped with filter paper and the halochromic properties were determined by recording the UV–VIS spectra. The UV–VIS absorption spectra were registered on an Agilent Cary 60 spectrophotometer (Agilent Technologies, Waldbronn, Germany) at 25 °C.

#### 2.2.3. Evaluation of Antioxidant Activity/Capacity

The antioxidant properties of the dye-treated polymeric films were cut into 1 cm × 1 cm pieces and immersed in methanol (3 mL) and subjected to ultrasonication according to a procedure previously described [43]. After 30 min 2.5 mL of solution were collected and transferred to plastic UV cells adding DPPH solution (1 mM, 0.5 mL). The samples were allowed to react for 30 min at room temperature for 30 min before measuring the absorbance at 517 nm. In addition, a control solution was prepared by mixing 2.5 mL methanol with 0.5 mL DPPH. The antioxidant activity was calculated using the equation:

$$AA\left[\%\right]=\frac{A_{sample}}{A_{DPPHi}}\times 100$$

#### 2.2.4. Evaluation of the Moisture Content

Using a method described by Zhang J. et al., to evaluate the water content, the polymeric films cut into 1 cm × 1 cm pieces, were weighed (*M*_1_), and dried at 105 °C in an oven until the mass was constant (*M*_2_) [40]. 

The water content was calculated using the equation:

$$Moisture\;content\;[\%]=\frac{M_{1}-M_{2}}{M_{1}}\times 100$$

#### 2.2.5. Evaluation of Swelling Capacity and Solubility

The swelling capacity and solubility were tested using a method which was previously described by Lu R. et al. 1 cm × 1 cm pieces of films were placed in an oven at 105 °C until the mass was constant (*M*_1_) and were immersed in 30 mL of distilled water in closed vessels at 25 °C for 24 h. After this time, the films were carefully drained with filter paper and weighed (*M*_2_). The samples were then placed in an oven at 105 °C to dry until the weight was constant (*M*_3_) [41]. 

The swelling capacity and water solubility were calculated using the following equation:

$$Swelling\;capacity\;\left[\%\right]=\frac{M_{1}-M_{2}}{M_{1}}\times 100$$

$$Water\;solubility\;[\%]=\frac{M_{3}-M_{1}}{M_{1}}\times 100$$

#### 2.2.6. Indicator Film Testing on Food Products

The sensitivity of indicator films for quality changes in food was tested in two circumstances: direct contact and contact with only the packaging atmosphere, on two food matrices: refrigerated chicken and pork meat, at three temperatures: 4 °C, 20 °C, and 40 °C.

To evaluate the sensitivity of the films to food quality changes, 1 cm × 1 cm pieces were placed in direct contact with meat samples weighing 10 g, then packed in plastic foil. Part of the samples was placed in the oven at 40 °C, another part was kept at 20 °C, and the third part was kept refrigerated at 4 °C. All samples were kept at the mentioned temperatures for 24 h.

In the case of contact with the packaging atmosphere, the experimental approach was similar with respect to the time to keep the samples at the specified temperatures, the difference being the type of packaging. Film sections were placed in Petri vessels and pieces of meat (10 g) were placed next to them, without having any contact. Subsequently, the Petri vessels were sealed with plastic foil. 

UV–VIS spectra were recorded for all samples at the initial moment and after 24 h of storage at the stated temperature.

#### 2.2.7. Film Characterization by Infrared Spectroscopy (ATR FT-IR)

FT-IR spectra were recorded on a Bruker Vertex 70 (Bruker Daltonik GmbH, Bremen, Germany) spectrometer equipped with a Platinium ATR, Bruker Diamond Type A225/Q, in attenuated total reflectance (ATR) mode. The spectra of the flavylium dye and the polymeric films were obtained through the co-addition of 64 scans in the spectral domain of 4000 to 400 cm^−1^ with a resolution of 4 cm^−1^.

#### 2.2.8. Thermogravimetric Analysis

A TG 209 F1 Libra thermogravimetric analyzer (NETZSCH-Gerätebau GmbH, Selb, Germany) was used to record the thermograms of the films and dye. The analyses were carried out in a nitrogen atmosphere from 20 to 600 °C, with a heating rate of 10 K/min. All data were processed with the Netzsch Proteus Thermal Analysis software version 6.1.0. (NETZSCH-Gerätebau GmbH, Selb, Germany). 

## 3. Results and Discussion

**Figure 1:**
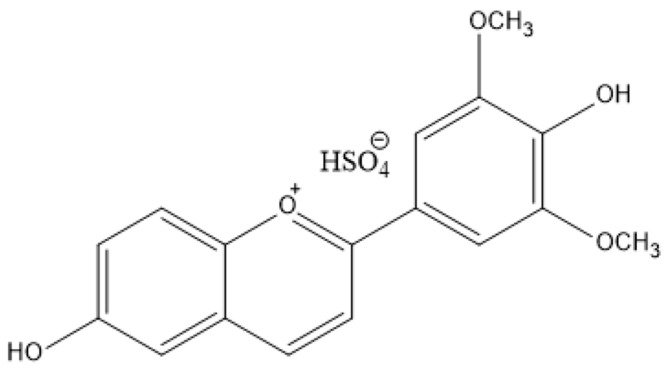
6-Hydroxy-4′-hydroxy-3′,5′-dimethoxyflavylium hydrogensulfate, the bio-inspired flavylium dye used in this work as a pH indicator for food quality monitoring.

The flavylium dye selected for this study was 6-hydroxy-4′-hydroxy-3′,5′-dimethoxyflavylium hydrogensulfate (chemical structure in Figure 1), previously synthesized and characterized by our group [38]. The rationale for incorporating this anthocyanin-based dye into such polymer matrices was the structural analogy with the natural anthocyanins, taking advantage of its pH-sensitive color changes, thus facilitating real-time monitoring of food product freshness. Chicken and pork meat matrices were used to test the effectiveness of these bio-based pH-sensitive indicators. The concentration of the flavylium dye in the polymer matrix was optimized by preliminary experiments, since higher concentrations could impede the monitoring of the real effects of dye embedding.

For film production, chitosan, PLA, and polyvinyl alcohol (PVA) were selected as polymer matrices due to their compatibility with biological materials, ability to form thin films, and food safety. In addition to its biodegradability and antimicrobial properties, chitosan is suitable for extending the shelf life of perishable foods [23]. On the other hand, PVA contributes to the mechanical strength and flexibility of the film, ensuring its durability and further applications in practical packaging [28]. PLA has excellent mechanical properties, transparency, and high strength, being already used in various packaged products [22,41]. 

### 3.1. Preparation and Characterization of the Polymer Films Loaded with Anthocyanin-Inspired Dye 

The films were prepared by solvent casting method and were characterized using spectroscopic methods (FT-IR and UV–VIS) and thermogravimetry (TG).

#### 3.1.1. Validation of Dye Incorporation into the Polymer Matrix by FT-IR and UV–VIS Spectroscopy

**Figure 2:**
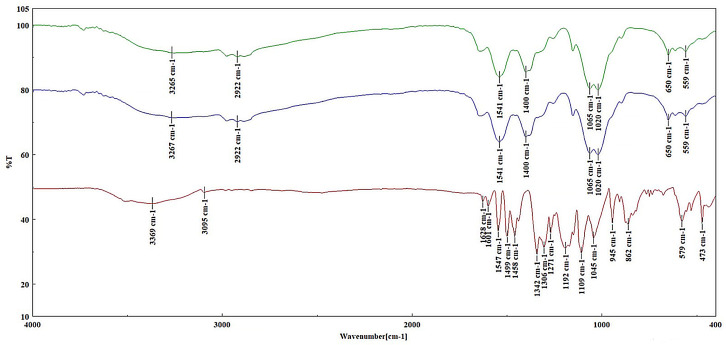
FT-IR spectra of chitosan (green), chitosan–dye (blue), and dye (red).

FT-IR spectroscopy was used to confirm the incorporation of the dye into the chitosan and PVA matrices. The overlaid FT-IR spectra of the chitosan–dye composite, as well as of the raw chitosan and dye are shown in Figure 2. The chitosan–dye film showed the following absorption characteristics bands assigned to chitosan and dye: 3267 cm^−1^ $\;(\nu_{{NH}_{2}})$, 2922 cm^−1^ ${\;(\nu }_{{CH}_{2}}^{as})$, 1541 cm^−1^ ($\delta_{{NH}_{2}}$), 1020 cm^−1^ ($\nu_{C-O}$). 

From the FT-IR spectra of the chitosan–PVA–dye film, the following characteristic absorption bands that are shifted from the absorption bands of the chitosan–PVA film and of the dye were identified and assigned: 3273 cm^−1^ ($\nu_{OH}$), 2909 cm^−1^ ($\nu_{{CH}_{2}}^{s}$), 1653 cm^−1^ ($\nu_{C=O}—$amide I-chitosan), 1560 cm^−1^ ($\delta_{{NH}_{2}}$) and $\nu_{C-N}$ overlapping), 1086 cm^−1^ ($\nu_{coc}^{as}$) (Figure S1). 

The FT-IR spectrum of the PVA–dye films showed absorption bands characteristic of the vibrational groups found in PVA and in the dye, which were assigned as follows: 3271 cm^−1^ $(\nu_{OH}$), 2939 cm^−1^ ($\nu_{{CH}_{2}}^{as}$), 2914 cm^−1^ ($\nu_{{CH}_{2}}^{s}$), 1086 cm^−1^ ($\nu_{coc}^{as}$) (Figure S2). 

The FT-IR spectra of the PLA–dye films showed the following absorption characteristic bands found in PLA and dye 2995 cm^−1^ ($\nu_{{CH}_{3}}^{as}$), 1749 cm^−1^ ($\nu_{C=O}$), and 1080 cm^−1^ ($\nu_{coc}^{as}$) (Figure S3).

In conclusion, the FT-IR analysis confirmed that the dye has been successfully incorporated into the matrix of each film. The specific absorption characteristic bands are consistent with the known values of the dye molecule, indicating the successful integration of the dye into the film matrices.

**Figure 3:**
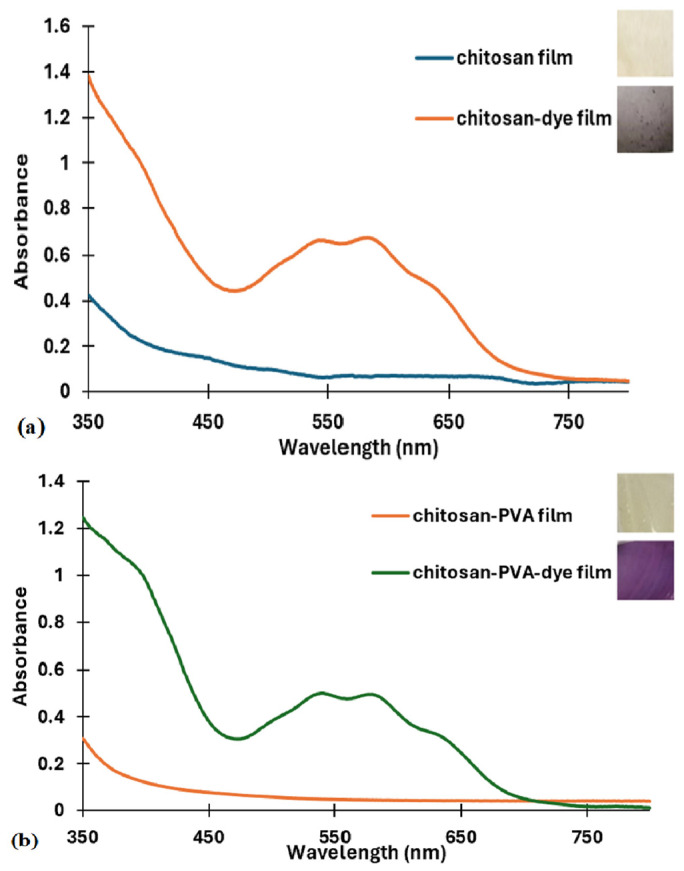

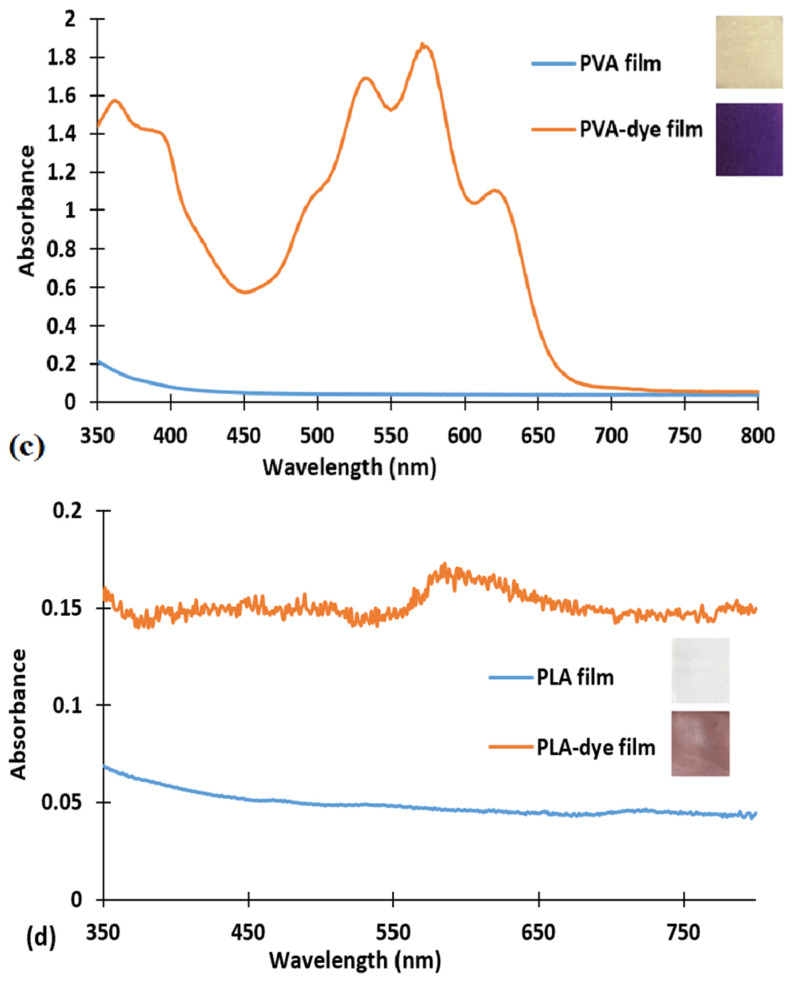
UV–VIS spectra of bio-based polymeric films (control and with dye): (**a**) chitosan, (**b**) chitosan–PVA, (**c**) PVA, (**d**) PLA.

The incorporation of the dye into the polymer matrices and the transparency of the films were also demonstrated by UV–VIS spectroscopy. Following the preparation of the films according to the described methods, they were dried. The evaluation was carried out under the same conditions as that of a film in the absence of the dye. Figure 3 shows that the bio-based polymer films with added dye, with the exception of the PLA film, exhibited specific absorption maxima in the visible region, while the control films showed no significant absorption.

Compared to the dye, which had an absorption maximum of 490 nm, the chitosan–dye and the chitosan–PVA–dye composite films (Figure 3a,b) showed an absorption maximum at about 580 nm, whereas in the case of the PVA–dye film (Figure 3c), the maximum appeared at 575 nm. The PLA–dye film was highly pigmented, meaning that an absorption maximum could not be observed (Figure 3d).

Two shoulders could be observed at 540 nm and 640 nm for both chitosan–dye and chitosan–PVA–dye films, while for the PVA–dye film, the shoulders were detected at 540 nm and 625 nm.

#### 3.1.2. Thermal Stability and Performance of Dye-Based Films

**Figure 4:**
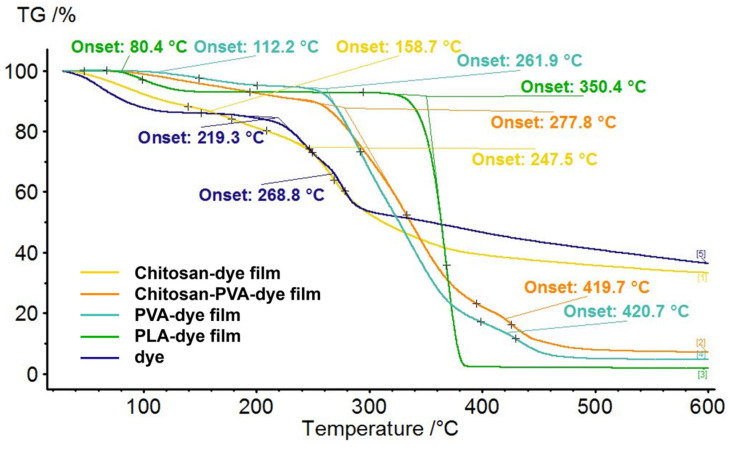
Thermograms of the chitosan–dye film (yellow), chitosan–PVA–dye film (orange), PVA–dye film (turquoise), PLA–dye film (green), and dye (indigo).

The thermal properties of the bio-based polymeric films were analyzed using thermogravimetric methods to assess their stability and decomposition temperature. Figure 4 shows the thermograms of the dye and all bio-based polymeric films obtained with and without dye. It can be observed that the thermograms of PVA–dye composites show three stages of weight loss, whereas the thermogram of the dye is similar to that of the chitosan–dye film, with two weight loss stages. For the chitosan–PVA–dye and PLA–dye composites the thermograms reveal two stages of weight loss, similar to the case of the flavylium dye. However, for these composite films, the second decomposition stage started at 419.7 °C and 346.6 °C, respectively, at a much higher temperature than for the dye (249.3 °C).

Comparing the thermograms of the chitosan–PVA, the PVA and the PLA films with and without the dye with the thermogram of the bio-inspired dye, it can be observed that up to 300 °C the films were more stable than the dye.

Regarding the weight loss, Table 1 shows that for three films, chitosan–dye, PVA–dye, and PLA–dye, the weight loss in the first step was less than 5%, probably due to some water content. The chitosan–PVA–flavylium dye composite presented a major weight loss (about 40%) already in the first thermal decomposition stage that occurred in the temperature range of 190–330 °C. The highest weight loss (67.09%) was observed for the PLA–dye film in the second thermal decomposition step that occurred in the temperature range of 290–385 °C, denoting better thermal stability compared to the other tested films. At the end of the analysis, the chitosan–dye film and the raw dye presented the highest amounts of residue, while in the case of the other films the residue was less than 10%. 

### 3.2. Sensitivity of the Films to pH Changes

The sensitivity of the films with the incorporated dye was evaluated by immersion in 10 mL of buffer solutions with pH values ranging from 2 to 12, in 10 steps for chitosan and 11 steps for the other three matrices. After one hour of immersion, the UV–VIS spectra of the films were registered to evaluate the displacements of absorption maxima and the color changes. 

**Figure 5:**
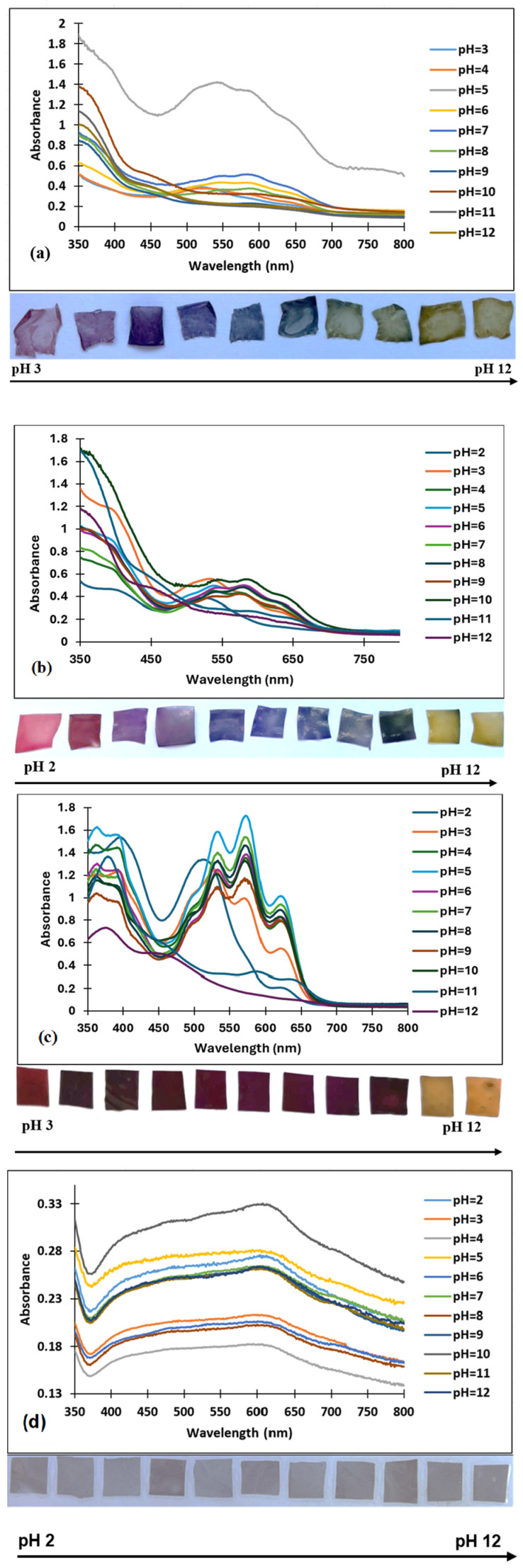
UV–VIS spectra for: (**a**) chitosan–dye films; (**b**) chitosan–PVA–dye films; (**c**) PVA–dye films, (**d**) PLA–dye films.

Due to the high solubility of the chitosan under acidic conditions, the chitosan-based film was degraded at pH value 2. For these films small changes in the absorption maxima were observed in the following pH ranges: from 5 to 6 and from 8 to 9, which were macroscopically visible (Figure 5a).

In the case of the chitosan–PVA films, five stages of color change were observed (Figure 5b) through more pH ranges: reddish pink (pH = 2–3), purple (pH = 4–5), blue (pH = 6–9) and yellow (pH = 11–12), a fact that is also reflected by the presence of more absorption maxima in the UV–VIS spectra. 

PVA films did not show evident color changes, but in the pH range from 11 to 12 shifts of color and absorption maxima were observed (Figure 5c).

PLA films did not show significant color changes, a fact also demonstrated by the spectroscopic analysis (Figure 5d), as the absorption maxima at pH variations are absent.

As a result of the different pH ranges in which the films have changed color, the potential application may vary depending on the substrate/products in which they can come in contact.

### 3.3. Antioxidant Properties of the Polymer Film–Dye Composites

As the dye used had antioxidant activity, it was necessary to determine whether the films would also have this activity in order to be used as smart packaging.

As shown in Table 2, the polymeric–dye films did not show antioxidant activity, proving that the films had very low permeability and DPPH could not reach the dye, a possible explanation being the impossibility of releasing the dye from the polymeric matrix during the assay time. This firm keeping of the dye inside the polymer matrix means that leaching of the dye is not significant even in direct contact with the food and can also indicate the non-toxicity of the composite film despite a potential toxic effect of the dye itself at higher concentrations. However, the possible lack of cytotoxicity of the dye as well as of the composites must be undoubtedly demonstrated and will be the subject of a forthcoming work. 

### 3.4. Evaluation of the Moisture Content

As the results from Table 3 show, the chitosan–PVA–dye and PVA–dye films had a moisture content of less than 7%, while for the chitosan–dye and PLA–dye matrices, the moisture content reached 21%. These differences in the moisture content can be primarily attributed to the inherent hydrophilicity of the polymers. Chitosan and PLA, which are more hydrophilic, have higher moisture contents compared to those of PVA-containing films. Blending chitosan with PVA results in a film with reduced moisture content, indicating that the interaction between the two polymers modifies the overall hydrophilicity and moisture absorption properties. Consequently, for applications requiring lower moisture content PVA-based films (either PVA–dye or chitosan–PVA–dye) are more suitable. On the contrary, for applications where moisture retention is beneficial PLA–dye, or chitosan–dye films may be preferred.

### 3.5. Evaluation of Swelling Capacity and Solubility

As shown in Table 4, there were significant variations concerning the water solubility of the films, ranging from 0% to 87%, but these did not depend on the chemical structure or composition of the dye.

The swelling degree is raised in most of the films, except for the PLA–dye film, from which this property was not expected. This fact makes the chitosan–dye, chitosan–PVA–dye, and PVA–dye films suitable for detecting storage conditions of food in high humidity. Blending chitosan with PVA results in a film with reduced water solubility and high swelling capacity, suggesting a balance between the hydrophilic properties of chitosan and the water-absorbing nature of PVA. PVA–dye films offer moderate solubility and swelling, making them useful in areas where partial water interaction is required. PLA dye films, with zero solubility and swelling, are ideal for applications requiring water resistance and stability in aqueous environments.

### 3.6. Evaluation of the Efficacy of Film–Dye Composite Films as an Indicator of the Freshness of Samples of Food Products

All bio-based dye films were tested on pork and chicken meat to understand whether these films can be used as indicators in intelligent packaging.

**Figure 6:**
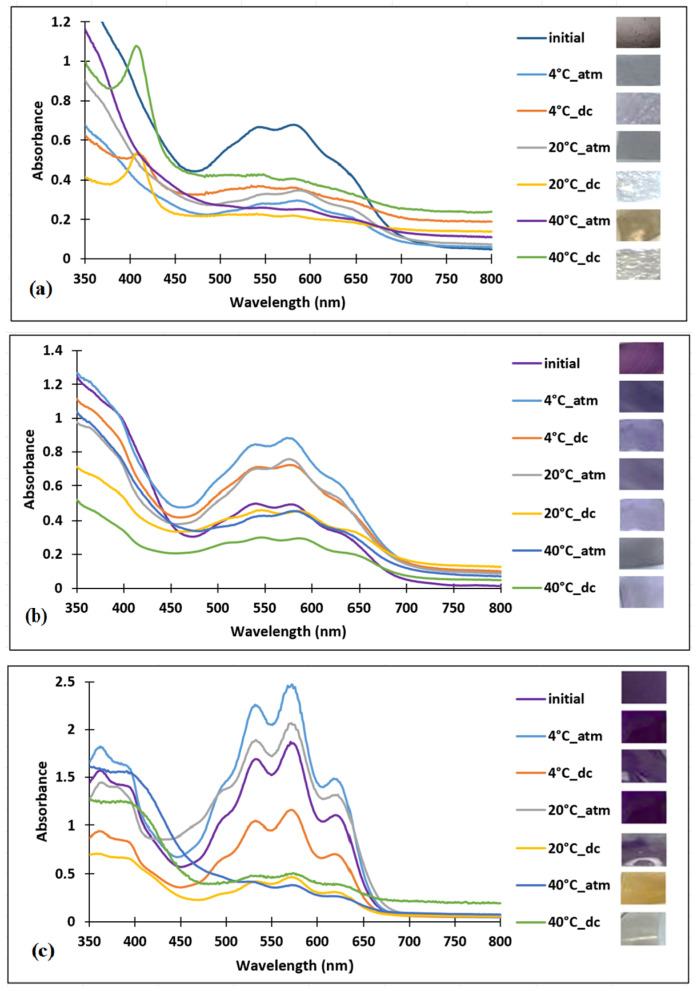

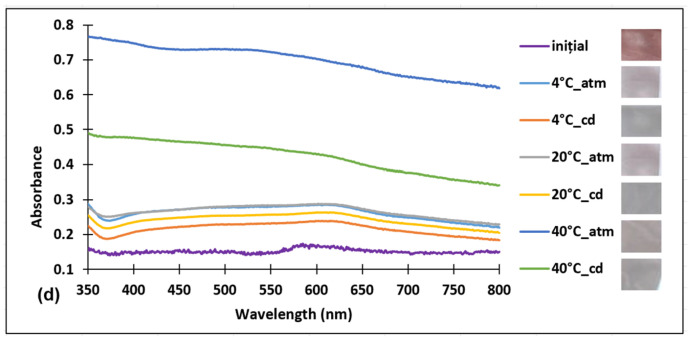
UV–VIS spectra of bio-based–dye polymers after 24 h of testing on pork meat (**a**) chitosan–dye, (**b**) chitosan–PVA–dye, (**c**) PVA–dye, (**d**) PLA–dye (dc-direct contact between film and meat, atm-contact between the film and packaging atmosphere).

For the first test, the films were kept in direct contact with pork meat and in contact with the packaging atmosphere at three different temperatures: 4 °C, 20 °C, and 40 °C, as seen in Figure 6. In addition, the films were incubated in an air environment at 20 °C and 40 °C (as control samples), to demonstrate that the color changes occurring throughout the test conditions were due to temperature.

Chitosan–dye films that were in direct contact with the pork meat underwent a significant color intensity modification at 20 °C and 40 °C, while in the case of contact with the packaging atmosphere, the most obvious change was at 40 °C.

For the chitosan–PVA film, a decrease in color intensity was observed from 4 °C to 40 °C for both situations tested.

In the case of PVA–dye film the most significant color change was at 40 °C for both the direct contact and contact with the packaging atmosphere.

For the PLA–dye film color changes could not be observed, as shown in Figure 6d, which indicates lower sensitivity of the PLA–dye film in comparison with the others. This can be attributed to the hydrophobicity of PLA, which probably prevented the dye from interacting effectively with the aqueous environment and responding to any modification of the pH values of the food sample, thus inhibiting any observable color change.

**Figure 7:**
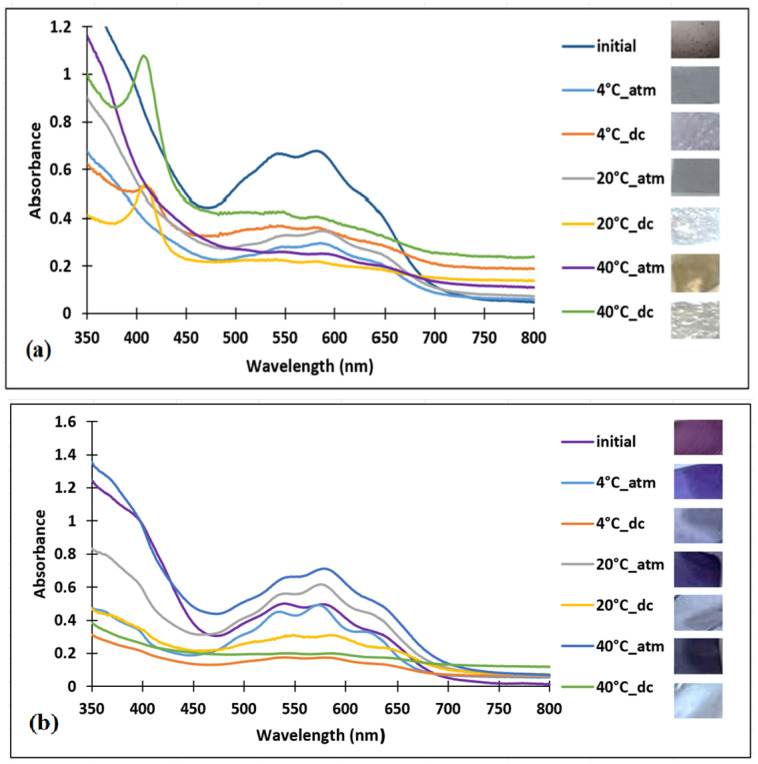

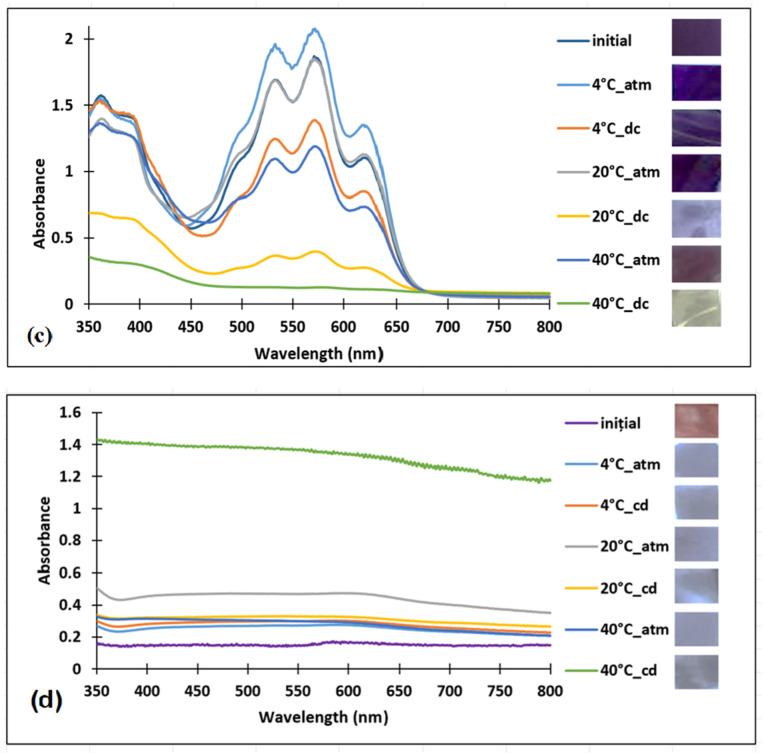
UV–VIS spectra of bio-based–dye polymers after 24 h of testing on chicken meat (**a**) chitosan–dye, (**b**) chitosan–PVA–dye, (**c**) PVA–dye, (**d**) PLA–dye (dc-direct contact between film and meat, atm-contact between film and packaging atmosphere).

For the second test, refrigerated chicken meat was used under the same testing conditions, as shown in Figure 7.

Due to the increased water content of chicken meat, only the chitosan–dye films that were in contact with the packaging atmosphere could be analyzed, but the UV–VIS spectra did not indicate important changes.

The spectroscopic analysis of the chitosan–PVA–dye films showed significant changes at all analyzed temperatures and circumstances, with the mention that for the contact with the packaging atmosphere, the most obvious change was observed at 40 °C.

For the PVA–dye films (Figure 7c), the UV–VIS spectra in the temperature range from 4 °C to 40 °C indicated a decrease in color intensity in both test situations but were more visible in direct contact with chicken meat.

The spectroscopic analysis of the PLA–dye film did not show any visible changes, as can be observed in Figure 7d.

## 4. Conclusions

In this study, a bio-inspired flavylium dye synthesized in our laboratory was successfully incorporated into biodegradable intelligent structures based on chitosan, chitosan–PVA, PVA, and PLA. The effects of this type of composite material on the absorbance intensity of the developed films were thoroughly investigated. Three out of four new biopolymer–dye composite films demonstrated detectable color variations in the pH range of 4 to 8, essential for applying them as freshness indicators for intelligent packaging. At the same time, the antioxidant activity of the films was not detected, probably due to the impossibility of releasing the dye from the polymeric matrix. The pH indicator films were tested for the first time on pork and refrigerated chicken meat and the color changes were obvious, demonstrating that these films are able to be used as indicators in intelligent packaging. The practical tests on meat products confirmed their effectiveness, paving the way for their use in commercial packaging solutions with the aim of improving food safety and reducing waste.

## Figures and Tables

**Table 1:** Weight losses in different thermal decomposition stages for the chitosan–dye, chitosan–PVA–dye, PVA, and PLA films, and for the raw dye.

Compound	Weight Loss [%]			
	1st Stage	2nd Stage	3rd Stage	Final
Chitosan–dye	4.21	16.09	-	66.74
Chitosan–PVA–dye	40.76	6.81	-	92.92
PVA–dye	2.48	21.88	5.48	95.32
PLA–dye	2.86	57.09	-	98.17
Dye	11.83	12.96	-	63.65

**Table 2:** Antioxidant activity of the polymeric films.

Polymeric Film	AA [%]
Chitosan–dye	99.92 ± 1.78
Chitosan–PVA–dye	99.82 ± 2.07
PVA–dye	99.13 ± 3.21
PLA–dye	95.72 ± 2.86

**Table 3:** Moisture content of the investigated polymeric films.

Polymeric Film	Moisture Content [%]
Chitosan–dye	17.20 ± 2.97
Chitosan–PVA–dye	6.66 ± 1.89
PVA–dye	6.26 ± 3.45
PLA–dye	20.80 ± 3.32

**Table 4:** Swelling capacity and solubility of the polymeric films.

Polymeric Film	Water Solubility [%]	Swelling Capacity [%]
Chitosan–dye	86.98 ± 4.13	-
Chitosan–PVA–dye	59.46 ± 3.65	81.76 ± 4.76
PVA–dye	52.72 ± 3.72	64.95 ± 3.77
PLA–dye	0.00 ± 0.00	0.00 ± 0.00

## Data Availability

Data are contained within the article and the Supplementary Material.

## Supplementary Materials

The following supporting information can be downloaded at: https://www.mdpi.com/article/10.3390/polym16162256/s1, Figure S1: FT-IR spectra of chitosan–PVA (green), chitosan–PVA–dye (blue) and dye (red); Figure S2: FT-IR spectra of PVA (green), PVA–dye (blue) and dye (red); Figure S3: FT-IR spectra of PLA (green), PLA–dye (blue) and dye (red); Figure S4: Thermograms of (a) chitosan (violet), chitosan–dye (yellow), and dye (indigo); (b) chitosan–PVA (blue), chitosan–PVA–dye (orange), and dye (indigo); (c) PVA (pink), PVA–dye (turquoise), and dye (indigo), (d) PLA (red), PLA–dye (green), and dye (indigo); Table S1: Weight losses on different temperature ranges for chitosan–dye film, chitosan film, and dye; Table S2: Weight losses on different temperature ranges for chitosan–PVA–dye film, chitosan–PVA film and dye; Table S3: Weight losses on different temperature ranges for PVA–dye film, PVA film, and dye.
