# Supplementary material for: Intelligent Biopolymer-Based Films: Promising New Solutions for Food Packaging Applications
Source: Polymers (Basel). 2024 Aug 8;16(16):2256. doi: 10.3390/polym16162256 (PMC11359790; doi:10.3390/polym16162256)
Supplement: Supplementary file 1 [file polymers-16-02256-s001.zip › polymers-3100761-supplementary.pdf]

Supplementary Materials

Intelligent Biopolymer-Based Films: Promising New Solutions for Food Packaging Applications

Diana Ionela Dăescu, Diana Maria Dreavă, Anamaria Todea, Francisc Peter and Iulia Maria Păușescu *

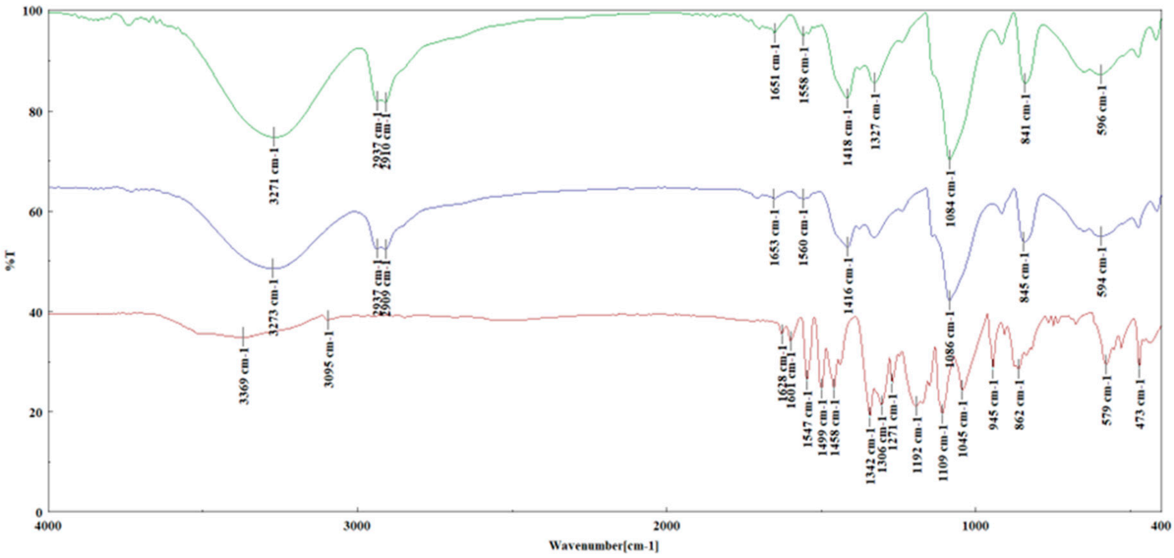

Figure S1. FT-IR spectra of chitosan-PVA (green), chitosan-PVA-dye (blue) and dye (red).

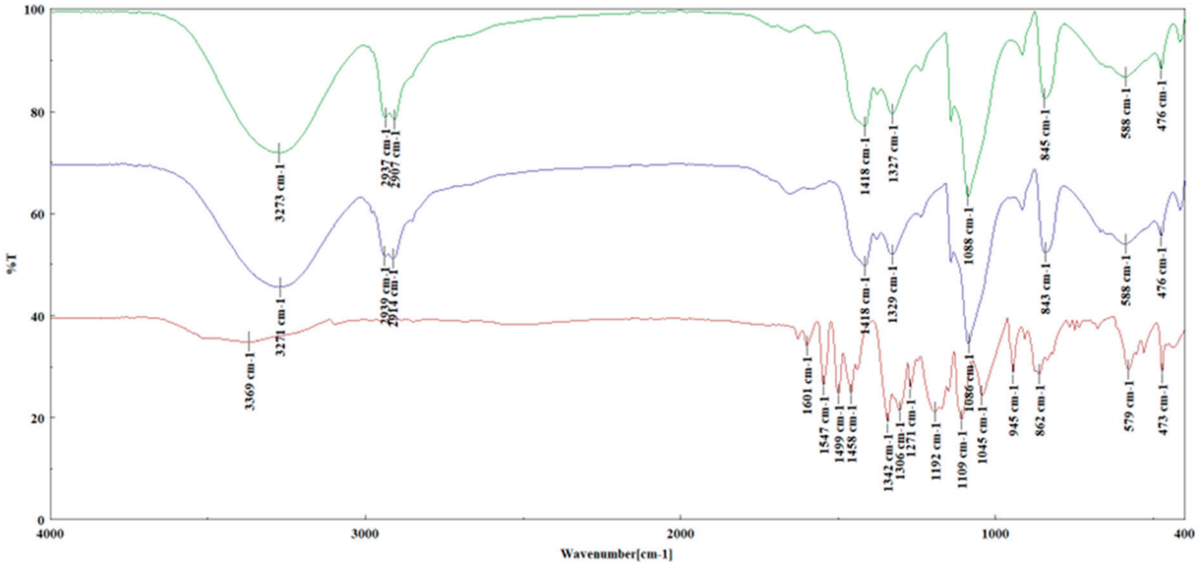

Figure S2. FT-IR spectra of PVA (green), PVA-dye (blue) and dye (red).

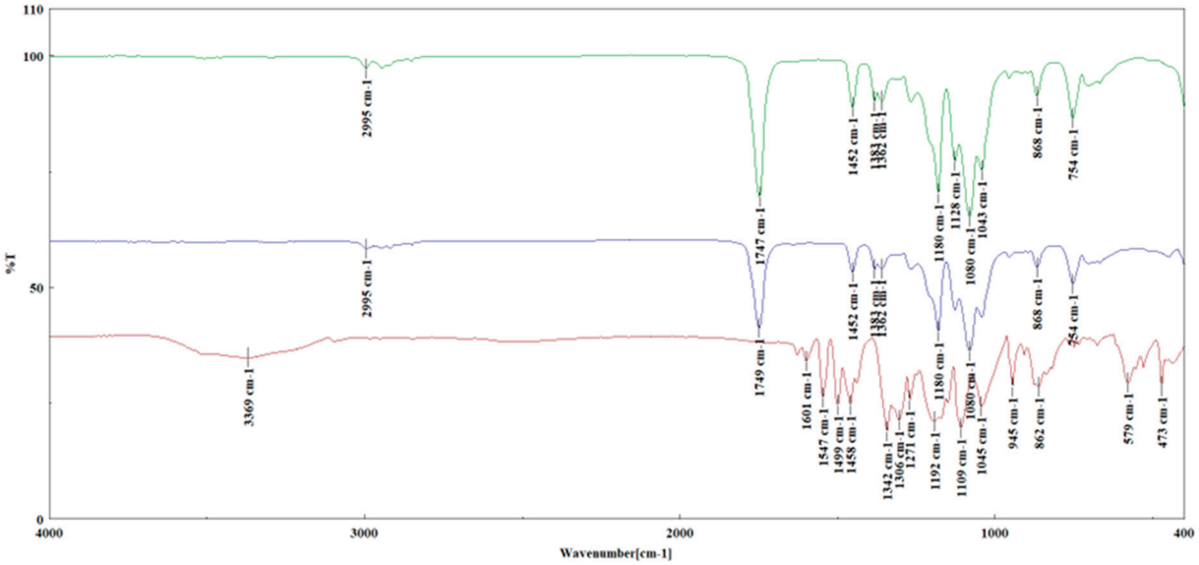

Figure S3. FT-IR spectra of PLA (green), PLA-dye (blue) and dye (red).

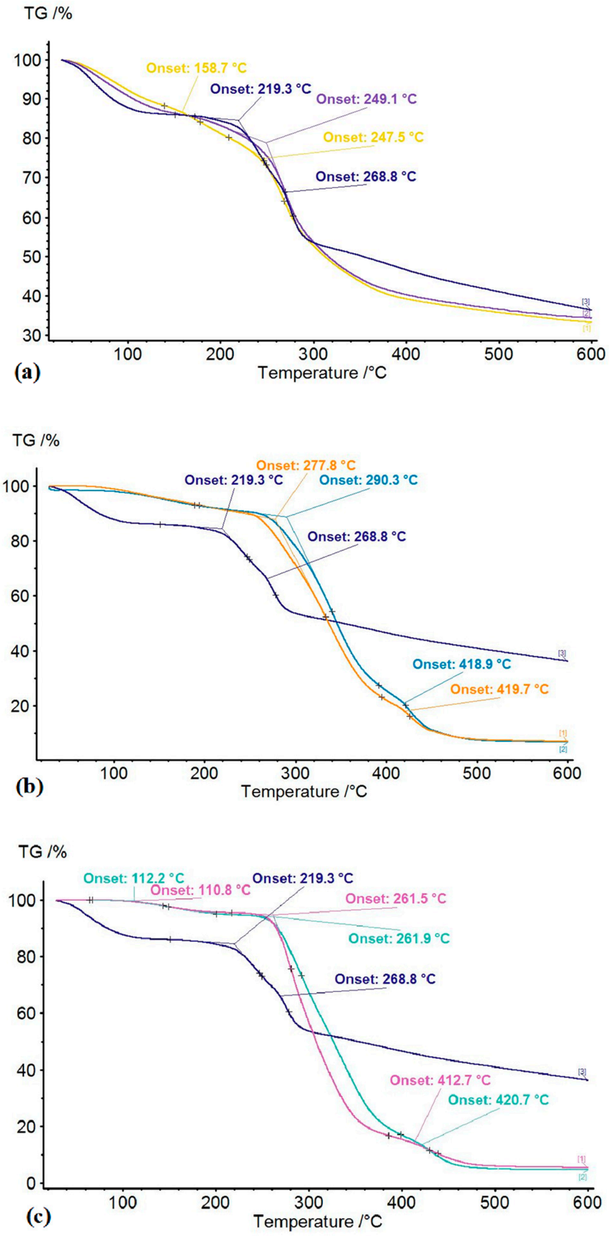

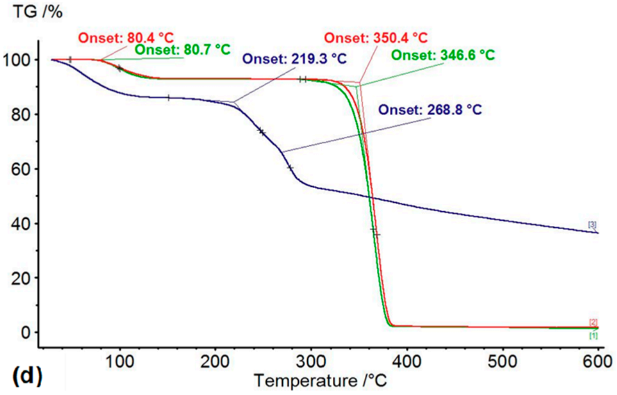

Figure S4. Thermograms of: (a) chitosan (violet), chitosan-dye (yellow), and dye (indigo); (b) chitosan-PVA (blue), chitosan-PVA-dye (orange), and dye (indigo); (c) PVA (pink), PVA-dye (turquoise), and dye (indigo), (d) PLA (red), PLA-dye (green), and dye (indigo).

Table S1. Weight losses on different temperature ranges for chitosan-dye film, chitosan film and dye.

Compound	Weight loss [%]				
	25-200°C	25-300°C	25-400°C	25-500°C	25-600°C
chitosan-dye	18.83	47.30	60.81	64.28	66.74
chitosan	16.82	46.38	59.73	63.47	65.71
dye	15.65	46.44	53.37	58.97	63.65

Table S2. Weight losses on different temperature ranges for chitosan-PVA-dye film, chitosan-PVA film and dye.

Compound	Weight loss [%]				
	25-200°C	25-300°C	25-400°C	25-500°C	25-600°C
chitosan-PVA-dye	7.43	28.79	77.91	92.09	92.92
chitosan-PVA	7.63	22.12	74.45	92.39	93.14
dye	15.65	46.44	53.37	58.97	63.65

Table S3. Weight losses on different temperature ranges for PVA-dye film, PVA film and dye.

Compound	Weight loss [%]				
	25-200°C	25-300°C	25-400°C	25-500°C	25-600°C
PVA-dye	4.80	32.64	83.11	95.04	95.32
PVA	4.30	43.23	84.65	94.07	94.53
dye	15.65	46.44	53.37	58.97	63.65
